# Analysis of the salivary microbiome using culture-independent techniques

**DOI:** 10.1186/2043-9113-2-4

**Published:** 2012-02-02

**Authors:** Vladimir Lazarevic, Katrine Whiteson, Nadia Gaïa, Yann Gizard, David Hernandez, Laurent Farinelli, Magne Østerås, Patrice François, Jacques Schrenzel

**Affiliations:** 1 Genomic Research Laboratory, Division of Infectious Diseases, Geneva University Hospitals, Rue Gabrielle-Perret-Gentil 4, CH-1211 Geneva 14, Switzerland; 2 Fasteris, Chemin du Pont-du-Centenaire 109, Case postale 28, CH-1228 Plan-les-Ouates, Switzerland

## Abstract

**Background:**

The salivary microbiota is a potential diagnostic indicator of several diseases. Culture-independent techniques are required to study the salivary microbial community since many of its members have not been cultivated.

**Methods:**

We explored the bacterial community composition in the saliva sample using metagenomic whole genome shotgun (WGS) sequencing, the extraction of 16S rRNA gene fragments from metagenomic sequences (16S-WGS) and high-throughput sequencing of PCR-amplified bacterial 16S rDNA gene (16S-HTS) regions V1 and V3.

**Results:**

The hierarchical clustering of data based on the relative abundance of bacterial genera revealed that distances between 16S-HTS datasets for V1 and V3 regions were greater than those obtained for the same V region with different numbers of PCR cycles. Datasets generated by 16S-HTS and 16S-WGS were even more distant. Finally, comparison of WGS and 16S-based datasets revealed the highest dissimilarity.

The analysis of the 16S-HTS, WGS and 16S-WGS datasets revealed 206, 56 and 39 bacterial genera, respectively, 124 of which have not been previously identified in salivary microbiomes. A large fraction of DNA extracted from saliva corresponded to human DNA. Based on sequence similarity search against completely sequenced genomes, bacterial and viral sequences represented 0.73% and 0.0036% of the salivary metagenome, respectively. Several sequence reads were identified as parts of the human herpesvirus 7.

**Conclusions:**

Analysis of the salivary metagenome may have implications in diagnostics e.g. in detection of microorganisms and viruses without designing specific tests for each pathogen.

## Background

The microbiota in the mouth has a significant impact on both the oral and general health. Bacterial species associated with periodontal health and those that are more prevalent in periodontal disease have been identified [1]. The salivary microbiota is a potential diagnostic indicator of several diseases. For instance, a caries-free oral status in children is associated with a significant shift in the relative abundance of *Porphyromonas catoniae *and *Neisseria flavescens *in saliva [2]. Increased salivary counts of *Capnocytophaga gingivalis*, *Prevotella melaninogenica *and *Streptococcus mitis *are associated with oral cancer [3]. The salivary level of the bacterium *Selenomonas noxia *correlates with obesity in women [4].

The study of the oral microbiota as well as its salivary component requires culture-independent techniques, since about one third of 700 bacterial species identified in the human oral cavity have not been cultivated [5]. These may be based on PCR amplification and high-throughput sequencing of the bacterial 16S rRNA genes (16S-HTS) or the metagenomic whole genome shotgun (WGS) sequencing. The latter approach may include either the analysis of the totality of generated DNA fragments or of the 16S rRNA gene fragments retrieved from the metagenome (16S-WGS) [6]. Both 16S-WGS and 16S-HTS approaches present limitations and advantages over each other [6].

Here we explored the microbial community composition in the saliva sample using WGS, 16S-WGS and 16S-HTS. In addition, to assess putative biases due to PCR amplification, we compared taxonomic composition of 16S-HTS datasets obtained after different number of PCR cycles.

## Methods

### Sampling

The study was conducted according to the current version of Declaration of Helsinki and approved by the Ethics Committee of HUG (09-078). Unstimulated saliva was obtained with informed consent from a 32-year male smoker without obvious signs of oral disease. The sample was collected by spitting in a sterile plastic 50-mL tube at 10:30 a.m., 1.5 hours after eating. Six hundred μL saliva was mixed with the same volume of 2x lysis buffer [Tris 20 mM, EDTA 2 mM (pH 8), Tween 1%] and Proteinase K (Eurobio) 200 μg/mL. After a 2.5 hour incubation at 55°C, proteinase K was inactivated by a 10-min heating at 95°C. The saliva lysate was divided in six 200-μL aliquots to which RnaseA (Roche) 40 μg/mL was added. Samples were incubated for 5 min at room temperature. From that point, the DNeasy Blood & Tissue Kit (QIAGEN) was used following the manufacturer's Spin-Column Protocol for Purification of Total DNA from Animal Blood or Cells (DNeasy Blood & Tissue Handbook 07/2006). DNA was eluted using 110 μL of supplied AE Buffer, then the pooled eluate (metagenomic DNA) was concentrated to 80 ng/μL. Total DNA quantity was assessed using a NanoDrop ND-8000 spectrophotometer (NanoDrop Technologies).

### PCR and sequencing

PCR amplification was carried out in a 50-μL PrimeStar HS Premix (Takara) containing 8 ng of purified DNA and 0.5 μM of each forward and reverse primer. The 16S rDNA V1-3 amplicons generated with primers 5'-GAGTTTGATCMTGGCTCAG (V1 forward) and 5'-CCGCGRCTGCTGGCAC (V3 reverse) corresponded to *E. coli *positions 28 to 514 after exclusion of primers sequences. The samples were run in four replicate PCRs for 20, 25 or 30 cycles using the following parameters: 98°C for 10 s, 60°C for 15 s, and 72°C for 1 min. The four replicate PCRs were then pooled.

Paired-end DNA libraries were prepared according to the manufacturer's (Illumina) instructions. Metagenomic DNA fragments of about 300 bp and 16S rDNA amplicons were barcoded using specific 6-base sequences. The libraries were sequenced from both ends for 100 cycles (excluding barcode sequences) on the Illumina Hi-Seq 2000 using TruSeq SBS v5 kit. A barcoded PhiX reference was spiked in the same channel to estimate the error rate.

### Sequence filtering

Parameters of the initial quality filter were the following: (i) maximum one base below a quality of 5 in the first 70 bases; (ii) a minimum average quality of 10; (iii) no ambiguous base allowed. After filtering, the average Q30 was larger than 75% and the average PhiX error rate was 0.7%. Only pairs were retained in the filtered data, i.e. if one read was filtered out the paired read was removed. Each of the three 16S-HTS datasets (20, 25 and 30 PCR cycles) was reduced by randomly picking 1.2 million sequence read pairs. Then, in the second filtering step, we removed sequences containing incorrect PCR primer sequences or runs of ≥ 12 identical nucleotides. The WGS dataset was reduced to one million sequence pairs and was not subject to additional filtering steps. Sequences were deposited in MG-RAST under accession numbers 4477823.3, 4477824.3, 4477839.3, 4477840.3, 4478078.3, 4478079.3, 4478080.3, 4478370.3, 4478371.3, 4479520.3, 4479521.3, 4479522.3, 4479523.3 and 4479524.3.

### Analysis

The 16S rDNA sequences were clustered to operational taxonomic units (OTUs) defined at 95% identity using CD-HIT [7]. The V1 and V3 sequences were assigned the taxonomic identity using the Ribosomal Database Project (RDP) Classifier [8] with a recommended 50% confidence cutoff. Taxonomic assignments of sequences from the WGS dataset were made using BLASTN [9] against NCBI prokaryotic, viral and fungal databases as well as against the human sequences from NCBI and EBI databases. The criteria used were a wordsize of 16, ≥ 94% identity, ≥ 90 overlap and e-value ≤10^-30^. The bacterial 16S rDNA sequences were extracted from the WGS dataset using CAMERA [10] and HMMER search option. They were then filtered using an e value ≤10^-10 ^and assigned to genera using the RDP Classifier.

Group-average clustering of data was performed using a Bray-Curtis similarity matrix in PRIMER-E (Plymouth), based on square-root-transformed genera abundance.

## Results and Discussion

### Illumina sequencing

We explored the microbial community composition in the saliva sample from a male healthy adult using Illumina sequencing. The 100-base paired reads from the whole metagenome fragments as well as 81-base V1 and 84-base V3 reads of 16S rDNA amplicons were analyzed (Table 1). Data from the forward and reverse run for each pool of DNA fragments were analyzed separately since it has been reported that reverse Illumina reads are of lower quality than those from the forward run [11].

**Table 1:** Description of the 4 sequence datasets and 14 subsets

	Dataset						
	16S-HTS, 20 cycles		16S-HTS, 25 cycles		16S-HTS, 30 cycles		WGS
**Generated reads**	16,345,598		10,732,108		6,150,466		14'724,838
**Randomly chosen read pairs**	1,200,000		1,200,000		1,200,000		1,000,000
**16S rDNA region subset**	V1	V3	V1	V3	V1	V3	
**Filtered forward reads**	592,322	566,310	576,701	564,774	546,710	569,459	1,000,000
**Filtered reverse reads**	532,945	530,504	534,229	523,548	548,009	495,531	1,000,000

### Taxa abundance as a function of the PCR cycle number in the 16S-HTS datasets

**Figure 1:**
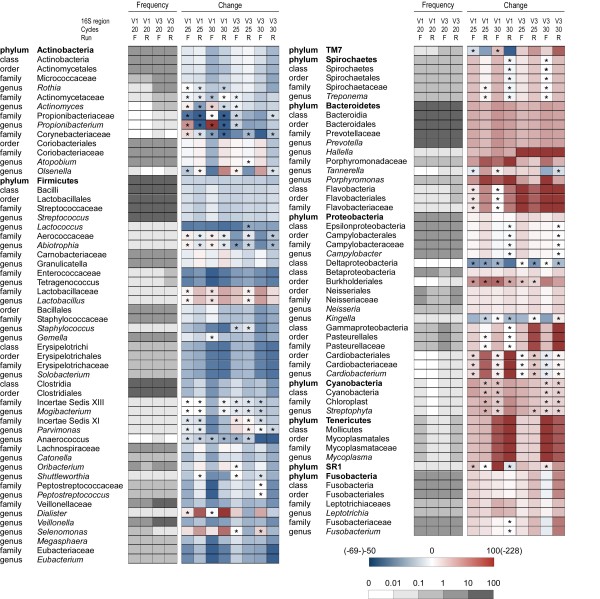
**Heat map showing changes in taxa proportions as a function of PCR cycle number**. The taxa shared by all (twelve) 16S-HTS subsets were analyzed. The relative abundances of taxa after 20 PCR cycles were used as baselines for comparisons. These values are represented according to the grey scale below the heat map. Changes (%) in the relative abundance of taxa after 25 or 30 cycles are represented by rectangles according to the color scale below the heat map. The corresponding values are given in the Additional files 1 and 3. Differences were significant (P < 0.01; chi-square test) unless marked by an asterisk.

Taxa detection and the accuracy of 16S rDNA abundance measurement are affected by the number of PCR cycles used to amplify 16S rDNA from a bacterial community [12-14]. To investigate how taxonomic composition of the same sample differed depending on the number of PCR cycles, we analyzed forward and reverse reads from V1 and V3 16S-HTS datasets obtained after 20, 25 and 30 cycles. The taxonomic assignment was performed using RDP Classifier (Additional file 1). The changes in proportions were relatively consistent for the different taxa within the same phylum (Figure 1): the relative abundance of sequence reads assigned to taxa belonging to the phyla Actinobacteria and Firmicutes generally decreased with more PCR cycles, whereas the proportion of sequences representing other phyla and their corresponding lower-level taxa generally increased. Instances in which the same direction of change (decrease or increase) occurred across all 8 subsets were found in 63 of the 109 taxa (Figure 1). Moreover, in 95% of cases where a > 25% change in taxa abundance was found in 30- vs 20- cycle-samples, the value obtained after 25 cycles was intermediate relatively to those obtained after 20 and 30 cycles. It seems likely that, for some taxa, more PCR cycles will further increase the bias. We found that the average Firmicutes to Bacteroidetes ratio, which may be an indicator of obesity in intestinal microbiota [15], was on average 3.6 (range 3.4-3.9), 2.3 (range 2.2-2.4) and 2.0 (range 1.9-2.2) in 20-, 25- and 30-cycle 16S-HTS datasets, respectively.

There is a concern that short Illumina reads and sequence errors may compromise the quality of taxonomic assignments [16-18]. To assess the accuracy of taxonomic assignments we extracted 81-base V1 and 84-base V3 sequences from the 16Sr RNA gene for 660 species from the Human Oral Microbiome Database (HOMD) [5] for which the taxonomic information was available at the genus level. These simulated Illumina reads were assigned taxonomy using the RDP Classifier with a recommended 50% bootstrap cutoff. The proportion of V1 and V3 sequences correctly assigned at the genus level reached 68% and 76%, respectively (Additional File 2). For both, V1 and V3 regions, the accuracy of taxonomic assignment at the phylum level was greater than 95%.

We clustered sequence reads generated by Illumina sequencing into OTUs, defined at ≥ 95% identity, which roughly corresponds to genus-level grouping [19] and may have the effect of absorbing some sequence errors [20]. Then, we compared the OTU content across datasets obtained after different number of PCR cycles using the phylum-level affiliation of representative OTUs derived from the RDP Classifier. The OTUs that met the criteria described in Additional file 3 were selected for comparisons. This approach confirmed the trend observed when taxonomy was assigned to each sequence read (see above); the relative abundance of the majority of OTUs from the phyla Actinobacteria and Firmicutes was decreased whereas the proportion of most OTUs from the phyla Bacteroidetes, Fusobacteria and Spirochaetes was enhanced by increasing the number of PCR cycles (Additional file 3).

Therefore, performing more PCR cycles, which may be required when little template DNA is available, may introduce amplification biases and increase the distance from samples for which less PCR cycles were performed.

### Taxonomic assignment in the WGS datasets

In the WGS approach, the taxonomic assignments were inferred from BLASTN searches of individual sequence reads. Sequences were compared to human genomic sequence as well as to databases containing completely sequenced prokaryotic, viral and fungal genomes. Most of the BLASTN hits corresponded to human DNA, whereas bacterial and viral sequences represented 0.73% and 0.0036% respectively (Table 2). Forward reads performed better in terms of assignment yield to these three categories, reflecting a higher sequence quality in comparison to the reverse reads [11]. A total of 369 and 367 16S rRNA gene fragments were extracted from WGS forward and reverse datasets, respectively, using CAMERA.

**Table 2:** Number of BLATSN hits against human, bacterial and viral databases

	BLASTN hit^a ^counts		R/F ratio
	F	R	
**Database**			
Human **^b^**	801677	642018	80.0
Bacteria **^c^**	7898	6807	86.7
Viruses **^d^**	38	34	89.5
Fungi **^e^**	0	0	-

F, forward run; R, reverse run.

**^a ^**The following BLASTN parameters were used: wordsize = 16, e ≤ 10^-30^, sequence identity ≥ 94%, alignment length ≥ 90 nt.

**^b ^**combined entries from ftp://ftp.ebi.ac.uk/pub/databases/fastafiles/emblrelease/em_rel_std_hum.gz as of 4 March 2011, and ftp://ftp.ncbi.nlm.nih.gov/blast/db/FASTA/human_genomic.gz as of 12 March 2011.

**^c ^**ftp://ftp.ncbi.nih.gov/genomes/Bacteria/ as of 16 November 2010.

**^d ^**ftp://ftp.ncbi.nih.gov/genomes/Viruses/ as of 25 November 2010.

**^e ^**ftp://ftp.ncbi.nih.gov/genomes/Fungi/ as of 3 March 2011.

### Comparison of the 16S-HTS and WGS datasets

**Figure 2:**
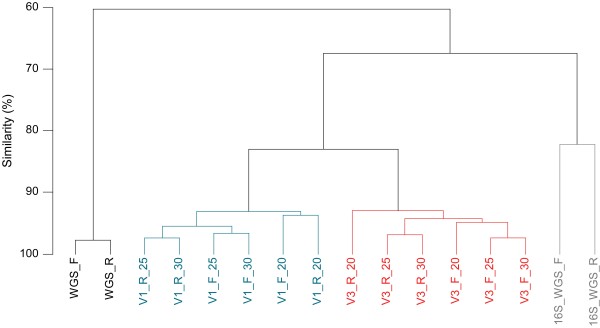
**Comparison of WGS, 16S-WGS and 16S-HTS datasets to study bacterial community of the saliva sample**. Group-average clustering of data was performed using a Bray-Curtis similarity matrix in PRIMER-E (Plymouth), based on square-root-transformed genera abundance. Only genera which occurred at a frequency of > 0.1% in at least one of 14 subsets were included in the analysis. V1 and V3 designate the sequenced hypervariable region of 16S rDNA. 20, 25 and 30 indicate the number of PCR cycles performed. F, forward reads; R, reverse reads.

We performed hierarchical clustering of WGS and 16S datasets based on the relative abundance of bacterial genera. Before computing the similarities, we applied a square-root-transformation of the relative abundance data in order to equilibrate the impact of abundant and rare genera. The resulting dendrogram (Figure 2) shows two main clusters that correspond to WGS and 16S-based datasets. The latter is divided into 2 subclusters corresponding to 16S-HTS and 16S-WGS approach. The 16S-HTS subcluster further splits according to the V region sequenced. Finally, for a given V region, datasets generated by sequencing PCR products obtained after 20 amplification cycles were separated from their 25- and 30-cycle counterparts which clustered together. The highest variation between the forward and reverse datasets was found for the 16S-WGS approach, which may be due to a rather small number (< 400) of sequences analyzed in comparison to the two other approaches (Table 1) and the fact that the forward and reverse reads in the 16S-WGS dataset cover different, randomly distributed segments of 16S rRNA genes which may lead to variation in taxonomic assignments.

In order to confirm that the pattern observed is reproducible across individuals, it would be necessary to analyze a larger number of salivary microbiomes using the same methodologies. As a first step towards addressing this issue, we extracted the 84-base V3 16S rDNA sequences from the 5 salivary microbiomes reported in a previous study [21]. The relevant data were then included in the construction of the similarity matrix as described above (not presented). We found that the average similarity between the V3 dataset determined in the current study and the five published microbiomes was comparable to the average similarity observed among these five microbiomes (Table 3). Relative to these values, our V3 16S-HTS datasets showed higher degrees of similarity when compared to our V1 16S-HTS and 16S-WGS datasets (Table 3) i.e. the interindividual differences may outweigh to some extent the methodological differences. Still, differences in the genera distribution inferred from 16S-WGS, 16S-HTS and WGS datasets (Table 3) are too great to allow for reliable comparisons of the results generated using different methodologies. However, as the reference microbial genome database will grow, the WGS and 16S-WGS methodologies will likely provide more closely related data.

**Table 3:** Similarity between the V3 16S and other datasets

Datasets to which the F and R 30-cycle V3 subsets were compared	Average similarity^a ^± SD (%)	Pearson's correlation coefficient (F, R)^b^
Itself (F vs R)	94.8	0.998
25-cycle V3	97.1 ± 0.3	0.998, 0.999
20-cycle V3	93.2 ± 1.5	0.974, 0.977
30-cycle V1	82.7 ± 0.8	0.987, 0.990
16S-WGS	68.5 ± 3.7	0.933, 0.958
WGS	64.1 ± 0.5	0.650, 0.652
V3 from other individuals^c^	64.8 ± 3.5	0.453-0.871, 0.458-0.867

F, forward run; R, reverse run.

^a ^The genera frequency were square-root transformed and used to construct the Bray-Curtis similarity matrix. When applicable, the similarities were calculated separately for the pairs of forward and the pairs of reversed subsets prior to averaging.

^b ^Based on untransformed data.

^c ^Samples 1.1, 2.1, 3.1, 4.1 and 5.1 from a previous study [21] in which the initial PCR cycles were carried out in the same conditions as those in the present work. Then, 28 cycles were performed using bar-coded primers and the amplicons were sequenced by the Roche/454 platform. The average similarity observed among these five microbiomes was 65.2 ± 7.2%.

We identified 206 bacterial genera using 16S-HTS, 108 of which have not been previously found in salivary microbiomes using culture-independent techniques [18,21-24]. This was also the case with 19 out of 56 genera determined by WGS, and 6 out of 39 genera identified by 16S-WGS approach. The majority of the new salivary genera (116/124) were found at a frequency < 0.1%, and only 8 occurred at a frequency between 0.1 and 0.74% (Additional files 1 and 4). This suggests that the most abundant bacterial genera in the saliva of healthy subjects have probably already been identified. However, the inventory and dynamics of low-abundance-genera, whose identification requires a deeper sample coverage, remain largely unknown.

Using WGS sequencing, which, in contrast to the 16S-HTS method applied in this study, does not specifically target bacteria, we did not detect archaea in the saliva sample. This is not surprising since the only archaeon identified so far in the human oral cavity i.e. *Methanobrevibacter oralis*, was found in dental plaques associated with pathological processes [25]. BLASTN similarity search against fungal genomes of the NCBI database did not yield any significant hits. The reasons for this may be: (i) an inefficient disruption of fungal cells by the enzymatic procedure used to release DNA from bacteria; (ii) the presence of fungi in saliva under the detection level; (iii) the absence of the relevant fungal genomes in the database. So far, sequences of only six fungal genera (*Zygosaccharomyces, Penicillium, Gibberella, Saccharomyces, Aspergillus, Candida*), present in the oral cavity of healthy individuals [26], are available in public databases.

### Salivary viriome

Based on BLASTN comparisons to the NCBI virus database, we identified sequences possibly derived from three different eukaryotic viruses. The most abundant (Table 4) was human herpes virus 7 with 17 and 15 sequence reads in the forward and reverse WGS datasets, respectively. A sequence similar to porcine endogenous retrovirus and the virus of the green alga *Chlorella *were identified as well. Several sequences produced the best BLASTN hits to bacteriophages including *Streptococcus *phage SM1 and two enterobacterial phages, lambda and phiX174.

**Table 4:** Detection of viral sequences in the WGS dataset using BLASTN

	Number of hits ^a^	
	F	R
**Virus**		
Human herpesvirus 7	17	15
Porcine endogenous retrovirus E	1	-
*Paramecium bursaria *chlorella virus-1 FR483	1	1
**Phage**		
Enterobacteria phage lambda	12	11
Enterobacteria phage phiX174	6	7
*Streptococcus *phage SM1	1	-

F, forward run; R, reverse run.

**^a ^**The following BLASTN parameters were used: wordsize = 16, e ≤ 10^-30^, sequence identity ≥ 94%, alignment length ≥ 90 nt.

### Clinical applications

Metagenomics has the potential to serve as a viral and bacterial infection control strategy in clinical practice because it can discover known as well as new pathogens, and might soon replace many existing typing methods in diagnostics.

HTS of cDNA has already been successfully applied to the detection of new viral pathogens in human serum and liver as well as in the reconstruction of viral genomes [27-29]. Similarly, WGS of a patient's feces samples detected the bacterial pathogen *Campylobacter jejuni *during but not after an acute diarrheal episode [30].

At least six double-stranded DNA human herpes viruses (HHV) i.e. Herpes simplex virus 1, Epstein-Barr virus, cytomegalovirus and human herpesviruses 6, 7 and 8 have been detected in saliva using sensitive PCR assays [31]. These viruses are shed in saliva asymptomatically which could facilitate their transmission. Most human adults are infected with HHVs but the prevalence of some HHV is significantly higher in HIV-seropositive persons. In subjects with recurrent oral Herpes simplex virus 1 infections, two other HHVs, HHV-6 and HHV-7 were simultaneously present with a frequency of over 93% [31]. HHvs possibly contribute to periodontitis which, in turn, facilitates virus shed into saliva [32]. Recently, using a metagenomic approach Willner et al. [33] identified Epstein-Barr virus in a pool of oropharyngeal swabs from 19 individuals.

In our study WGS sequencing applied on the salivary metagenome allowed identification of sequences showing the best similarity to the human herpesvirus 7 as well as to the putative periodontopathic bacteria *Porphyromonas gingivalis*, *Treponema denticola *and *Aggregatibacter actinomycetemcomitans *[34].

The exact role of bacteria and viruses in periodontitis and other oral diseases is not elucidated. It has been hypothesized that bacteria and viruses cooperate to provoke the disease [35]. The detection of periodontopathic agents is important because periodontitis has been associated with other health problems such as cardiovascular diseases, premature delivery, rheumatoid arthritis and cancer [35].

Although a large fraction of DNA extracted from saliva corresponds to human DNA, we estimate that, at a coverage consisting of a hundred million sequences, which is the current capacity per channel on the Illumina platform, hundreds of thousands of bacterial sequences and thousands of viral and phage sequences may be identified. Therefore, detection of viruses by metagenomic sequencing is possible even without including filtration and concentration steps, although these procedures are effective in enriching the metagenomic samples for viral DNA [33]. In addition, tens of thousands of 16S rDNA sequences, free of amplification anomalies, may be extracted from huge WGS datasets and used to assess taxonomic composition of bacterial communities.

## Conclusions

Analysis of the salivary microbiome is not only of interest from a fundamental perspective, but may have implications in diagnostics e.g. in detection of viruses and microorganisms without including specific tests for each pathogen.

In our study, WGS sequencing compared to 16S-HTS generated a higher fraction of taxonomically unassigned non-human sequences because of the lack of homologs in sequence databases. Using relatively stringent BLASTN parameters about 19% and 35% of sequence reads remained taxonomically unassigned in the forward- and reverse-run WGS subsets, respectively. Nevertheless, the advantage of the WGS approach is that it allows assessment of not only bacterial but also viral (human viruses and phages) and possibly fungal and archaeal communities which undoubtedly play an important role in oral health or disease. In addition, an in-depth sequencing of a salivary metagenome may provide insights into gene functions and allow for reconstruction of the functional potential of a microbial population [36]. Functional assignments of sequences may be made for instance using CAMERA, CARMA3 [37] or MG-RAST [38], as it was recently shown for the supragingival dental plaque microbome [39]. The obtained sequences may be assigned to known functions and classified to major categories including, among others, virulence and resistance to antibiotics.

## List of abbreviations

HHV: human herpes virus; HTS: high throughput sequencing; OTU: operational taxonomic unit; WGS: whole genome shotgun.

## Competing interests

The authors declare that they have no competing interests.

## Authors' contributions

VL, KW, PF and JS contributed to the experimental design and drafting the manuscript. VL carried out laboratory procedures. MØ and LF performed high-throughput sequencing. VL, NG, YG and DH performed the bioinformatics and taxonomic analyses. All authors approved the final manuscript.

## Supplementary Material

Additional file 1 **Relative abundance of taxa inferred from 16S-HTS and 16S-WGS datasets**. This Excel file lists taxa and their relative abundance. The RDP Classifier with a ≥ 50% confidence cutoff was used to assign taxonomy. Click here for file

Additional file 2 **Accuracy of taxonomic assignments for 81-base V1 and 84-base V3 16S rDNA sequences**. This graph shows the accuracy of taxonomic assignments for 660 HOMD species from 118 genera as determined using the RDP Classifier with a 50% bootstrap threshold. Click here for file

Additional file 3 **Changes in taxa proportions inferred from 16S-HTS subsets as a function of PCR cycle number**. This is a Word file showing changes (%) in the relative abundance of taxa identified after 25 and 30 cycles of PCR. The values obtained after 20 PCR cycles were used as baseline for comparisons. (A) Taxa identified in all (twelve) 16S-HTS subsets were selected for the analysis. (B) 95%-ID OTUs present in six subsets of the specified V region at a frequency > 0.1% in at least one 20-cycle-subset are presented. Changes in the relative abundance of taxa after specified number of cycles: red, increase; blue, decrease. Click here for file

Additional file 4 **Relative abundance of taxa in the WGS dataset**. This is an Excel file with the taxonomic assignments performed using BLASTN and Bergey's taxonomy accessible via the RDP [40]. Positive BLASTN hits values (wordsize of 16, ≥ 94% identity, ≥ 90 overlap and e-value ≤ 10^-30^) were normalized so that they sum to 100%. In cases where two or more top BLASTN hits were identical, the lowest common ancestor was calculated. Click here for file
