# Supplementary figures and images for: Analysis of the salivary microbiome using culture-independent techniques
Source: J Clin Bioinforma. 2012 Feb 2;2:4. doi: 10.1186/2043-9113-2-4 (PMC3296672; doi:10.1186/2043-9113-2-4)

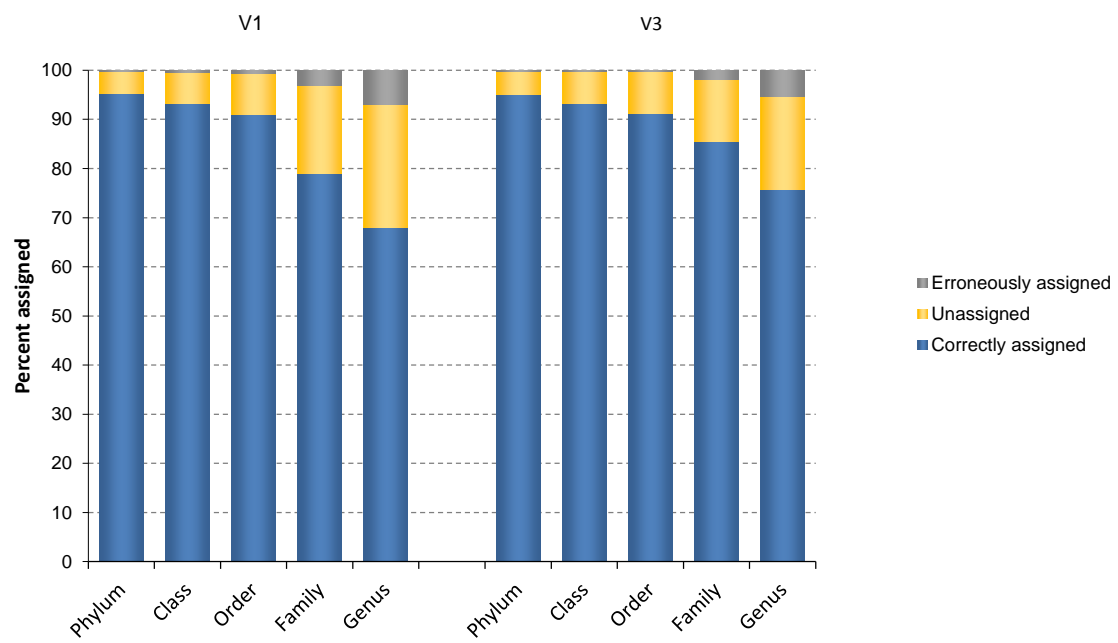

Supplement: Additional file 2 — Accuracy of taxonomic assignments for 81-base V1 and 84-base V3 16S rDNA sequences. This graph shows the accuracy of taxonomic assignments for 660 HOMD species from 118 genera as determined using the RDP Classifier with a 50% bootstrap threshold. [file 2043-9113-2-4-S2.PDF]
