# Supplementary material for: Analysis of the salivary microbiome using culture-independent techniques
Source: J Clin Bioinforma. 2012 Feb 2;2:4. doi: 10.1186/2043-9113-2-4 (PMC3296672; doi:10.1186/2043-9113-2-4)
Supplement: Additional file 3 — Changes in taxa proportions inferred from 16S-HTS subsets as a function of PCR cycle number. This is a Word file showing changes (%) in the relative abundance of taxa identified after 25 and 30 cycles of PCR. The values obtained after 20 PCR cycles were used as baseline for comparisons. (A) Taxa identified in all (twelve) 16S-HTS subsets were selected for the analysis. (B) 95%-ID OTUs present in six subsets of the specified V region at a frequency > 0.1% in at least one 20-cycle-subset are presented. Changes in the relative abundance of taxa after specified number of cycles: red, increase; blue, decrease. [file 2043-9113-2-4-S3.DOC]

**Additional file 3 - Changes in taxa proportions inferred from 16S-HTS subsets as a function of PCR cycle number**

A

|  |  |  | **Frequency (%)** | | | | **Change in taxa frequency^a^** | | | | | | | |  |  |
| --- | --- | --- | --- | --- | --- | --- | --- | --- | --- | --- | --- | --- | --- | --- | --- | --- |
|  |  |  | **F** | **R** | **F** | **R** | **F** | **R** | **F** | **R** | **F** | **R** | **F** | **R** | **Run** | |
|  |  |  | **V1** | **V1** | **V3** | **V3** | **V1** | **V1** | **V1** | **V1** | **V3** | **V3** | **V3** | **V3** | **V Region** | |
| **Rank** | **Taxon** |  | **20** | **20** | **20** | **20** | **25** | **25** | **30** | **30** | **25** | **25** | **30** | **30** | **Cycles** | |
| **phylum** | **Actinobacteria** | | 9.5890 | 7.7353 | 7.3006 | 8.7121 | -7.3 | -5 | -25 | -15.1 | -4.8 | -3 | -10.5 | -22.6 |  | |
| class | Actinobacteria | | 9.5890 | 7.7353 | 7.3006 | 8.7121 | -7.3 | -5 | -25 | -15.1 | -4.8 | -3 | -10.5 | -22.6 |  | |
| order | Actinomycetales | | 7.6134 | 6.4320 | 6.0227 | 6.7626 | -8.5 | -7.5 | -25.5 | -20.4 | -6.8 | -3.7 | -15.1 | -24.3 |  | |
| family | Micrococcaceae | | 0.4489 | 0.2957 | 5.0193 | 5.2150 | -8.5 | -10.1 | -24.1 | -14 | -6.5 | -4.1 | -13.7 | -27.9 |  | |
| genus | *Rothia* | | 0.0512 | 0.0229 | 5.0128 | 5.2098 | -0.3 | -6.8 | -17.4 | -14.7 | -6.4 | -4.1 | -13.7 | -28 |  | |
| family | Actinomycetaceae | | 0.0199 | 0.0126 | 0.4941 | 0.8222 | -3.4 | -6.2 | -19.2 | 0.2 | -4.2 | -9.1 | -18 | -12.9 |  | |
| genus | *Actinomyces* | | 0.0083 | 0.0062 | 0.4404 | 0.7316 | 9 | -30.5 | 23.8 | -11.6 | -4.3 | -10.1 | -16.1 | -13.4 |  | |
| family | Propionibacteriaceae | | 0.0034 | 0.0026 | 0.0388 | 0.0575 | -43.5 | -64.4 | 2.9 | -51.4 | -13.4 | -25.2 | -30.8 | -10.5 |  | |
| genus | *Propionibacterium* | | 0.0003 | 0.0004 | 0.0362 | 0.0547 | 54.1 | -50.1 | 225 | -51.4 | -13.4 | -29.4 | -28.2 | -15.8 |  | |
| family | Corynebacteriaceae | | 0.0024 | 0.0021 | 0.0101 | 0.0079 | 17.4 | -9.3 | -30.4 | -47 | -36.7 | -22.8 | -51.1 | -18.4 |  | |
| order | Coriobacteriales | | 1.8796 | 1.1907 | 1.1388 | 1.7474 | -2.6 | 7.3 | -24.3 | 13.8 | 5 | -2.9 | 14.2 | -18.8 |  | |
| family | Coriobacteriaceae | | 1.8796 | 1.1907 | 1.1388 | 1.7474 | -2.6 | 7.3 | -24.3 | 13.8 | 5 | -2.9 | 14.2 | -18.8 |  | |
| genus | *Atopobium* | | 1.7641 | 1.0729 | 1.0281 | 1.4920 | -3 | 6.7 | -24.8 | 10.8 | 4.5 | -1 | 14.6 | -16.6 |  | |
| genus | *Olsenella* | | 0.0184 | 0.0099 | 0.0074 | 0.0160 | -17.1 | 5.4 | -42.3 | 61.5 | 7.4 | -34.4 | 49.2 | -9.3 |  | |
| **phylum** | **Firmicutes** | | 58.5757 | 58.8640 | 62.7736 | 62.2855 | -13.8 | -13.9 | -14.9 | -15.4 | -13.6 | -15.7 | -15.2 | -19.3 |  | |
| class | Bacilli | | 39.8130 | 44.2284 | 45.6303 | 40.2855 | -10.2 | -14.8 | -6.5 | -14.4 | -14.6 | -12.8 | -13.7 | -11.7 |  | |
| order | Lactobacillales | | 37.6152 | 41.3881 | 44.4845 | 38.8097 | -10.3 | -14.8 | -6.6 | -14.4 | -14.5 | -12.7 | -13.2 | -11.2 |  | |
| family | Streptococcaceae | | 31.8273 | 34.8177 | 41.5205 | 34.8616 | -10.7 | -16.5 | -6.7 | -16.7 | -15 | -12.8 | -13.9 | -10.7 |  | |
| genus | *Streptococcus* | | 31.7414 | 34.6706 | 41.3597 | 34.6290 | -10.7 | -16.6 | -6.6 | -16.7 | -15 | -12.9 | -13.8 | -10.7 |  | |
| genus | *Lactococcus* | | 0.0169 | 0.0169 | 0.0178 | 0.0170 | -41.5 | -40.1 | -41.5 | -44.9 | -42.4 | -26.8 | -28.1 | -29.8 |  | |
| family | Aerococcaceae | | 0.0974 | 0.0854 | 0.0131 | 0.0138 | 8 | -5.3 | 8.2 | -5.5 | -32.2 | -7 | -35.5 | -17.9 |  | |
| genus | *Abiotrophia* | | 0.0945 | 0.0831 | 0.0111 | 0.0115 | 7.3 | -7.7 | 8 | -7.1 | -31.6 | -13.6 | -36.9 | -22.8 |  | |
| family | Carnobacteriaceae | | 3.6153 | 2.7554 | 2.7377 | 3.6147 | -5.9 | 7.5 | -5.3 | 15.3 | -6.3 | -12.3 | -3 | -16 |  | |
| genus | *Granulicatella* | | 3.6095 | 2.7410 | 2.7194 | 3.5781 | -5.9 | 7.4 | -5.3 | 15.2 | -6.4 | -12.3 | -3 | -16 |  | |
| family | Enterococcaceae | | 0.0947 | 0.0863 | 0.0895 | 0.1010 | -37.4 | -32.6 | -50.6 | -41.9 | -31.2 | -28.2 | -34.9 | -40.9 |  | |
| genus | *Tetragenococcus* | | 0.0930 | 0.0856 | 0.0894 | 0.1005 | -39.8 | -32.4 | -51.6 | -42.4 | -31 | -27.8 | -34.8 | -40.5 |  | |
| family | Lactobacillaceae | | 0.0471 | 0.0298 | 0.0314 | 0.0464 | 6 | 33 | 14.6 | 43.1 | 31.8 | 7.1 | 52 | 17.5 |  | |
| genus | *Lactobacillus* | | 0.0471 | 0.0298 | 0.0311 | 0.0449 | 5.7 | 33 | 14.6 | 43.1 | 29.9 | 7.3 | 48.6 | 19.2 |  | |
| order | Bacillales | | 1.4372 | 1.2063 | 1.1225 | 1.4330 | -7.3 | -12.5 | -3.7 | -19.9 | -20.4 | -17.4 | -32.4 | -26.5 |  | |
| family | Staphylococcaceae | | 1.4181 | 1.1817 | 1.1209 | 1.4305 | -7.3 | -13.4 | -3.8 | -19.7 | -20.5 | -17.5 | -32.8 | -26.6 |  | |
| genus | *Staphylococcus* | | 0.0550 | 0.0471 | 0.0636 | 0.0671 | -19.3 | -25.3 | -38.8 | -44.2 | -12.3 | -6.9 | -35.9 | -27.5 |  | |
| genus | *Gemella* | | 1.3624 | 1.1337 | 1.0568 | 1.3632 | -7.1 | -13 | -2.6 | -18.6 | -21 | -18 | -32.6 | -26.5 |  | |
| class | Erysipelotrichi | | 0.3930 | 0.3366 | 0.5140 | 0.5729 | -22.8 | -26.1 | -41.5 | -43.3 | -23.1 | -21.6 | -39 | -43.5 |  | |
| order | Erysipelotrichales | | 0.3930 | 0.3366 | 0.5140 | 0.5729 | -22.8 | -26.1 | -41.5 | -43.3 | -23.1 | -21.6 | -39 | -43.5 |  | |
| family | Erysipelotrichaceae | | 0.3930 | 0.3366 | 0.5140 | 0.5729 | -22.8 | -26.1 | -41.5 | -43.3 | -23.1 | -21.6 | -39 | -43.5 |  | |
| genus | *Solobacterium* | | 0.3555 | 0.3081 | 0.5119 | 0.5695 | -23.8 | -28 | -42.3 | -43.6 | -23.1 | -21.6 | -39.1 | -43.5 |  | |
| class | Clostridia | | 16.6691 | 12.3084 | 16.1565 | 20.7859 | -22.2 | -11.9 | -33.7 | -19.6 | -10.4 | -21.1 | -18.2 | -32.9 |  | |
| order | Clostridiales | | 15.4968 | 11.2830 | 15.9282 | 20.4969 | -22.4 | -11.9 | -33.8 | -19.8 | -10.4 | -21.1 | -17.9 | -32.7 |  | |
| family | Incertae Sedis XIII | | 0.0523 | 0.0385 | 0.0403 | 0.0520 | 0.4 | 0.7 | -24.2 | -3.2 | -7.2 | -8.6 | -14.1 | -18.2 |  | |
| genus | *Mogibacterium* | | 0.0523 | 0.0385 | 0.0403 | 0.0520 | 0.4 | 0.7 | -24.2 | -3.2 | -7.2 | -8.6 | -14.1 | -18.2 |  | |
| family | Incertae Sedis XI | | 0.0748 | 0.0574 | 0.0690 | 0.0916 | -8 | -9.7 | -37.1 | -20.5 | 11.8 | 5.3 | -11.5 | -17.4 |  | |
| genus | *Parvimonas* | | 0.0653 | 0.0501 | 0.0539 | 0.0775 | -5.5 | -5.8 | -38.1 | -20.6 | 18.7 | 8.2 | -2.2 | -12.5 |  | |
| genus | *Anaerococcus* | | 0.0027 | 0.0026 | 0.0102 | 0.0072 | -10.1 | -14.5 | -18.7 | -23.6 | -25.7 | -14.7 | -69.1 | -69 |  | |
| family | Lachnospiraceae | | 2.5971 | 1.4491 | 3.9830 | 6.5419 | -14.3 | 4.2 | -21.4 | 4.9 | -3.2 | -17.9 | -8.3 | -27.6 |  | |
| genus | *Catonella* | | 0.5166 | 0.3263 | 0.2460 | 0.4126 | -21.4 | -8.1 | -30.2 | -10.9 | -9.7 | -21.4 | -14.8 | -25.6 |  | |
| genus | *Oribacterium* | | 1.8571 | 0.9857 | 2.6727 | 4.4077 | -12.9 | 8.4 | -19.4 | 11.3 | -1.4 | -17.3 | -3.8 | -26.7 |  | |
| genus | *Shuttleworthia* | | 0.0741 | 0.0499 | 0.0560 | 0.0799 | -17.9 | -1 | -36.1 | -32.7 | 1.2 | -14 | -9 | -25 |  | |
| family | Peptostreptococcaceae | | 0.7167 | 0.3715 | 0.3496 | 0.7005 | -26.1 | -6.3 | -59.4 | -14.2 | -6.9 | -14.3 | 0.8 | -36.2 |  | |
| genus | *Peptostreptococcus* | | 0.7167 | 0.3713 | 0.3495 | 0.6999 | -26.1 | -6.3 | -59.5 | -14.4 | -6.9 | -14.2 | 0.8 | -36.2 |  | |
| family | Veillonellaceae | | 9.5364 | 7.3936 | 10.8919 | 12.3149 | -24.6 | -16.1 | -34.7 | -25.8 | -13.1 | -23.7 | -21.6 | -34.7 |  | |
| genus | *Dialister* | | 0.0539 | 0.0268 | 0.0212 | 0.0596 | 12.1 | 82.8 | -1.5 | 131.9 | 24.5 | -25.9 | 44.2 | -15 |  | |
| genus | *Veillonella* | | 8.1412 | 6.4622 | 10.1215 | 11.1339 | -26.5 | -18.7 | -35.9 | -29.8 | -13.7 | -24.2 | -22.3 | -35.6 |  | |
| genus | *Selenomonas* | | 0.2571 | 0.1389 | 0.0184 | 0.0390 | 16 | 51.1 | 14.8 | 73.2 | 2.2 | -25.6 | 22.4 | -22.4 |  | |
| genus | *Megasphaera* | | 0.6564 | 0.3895 | 0.6189 | 0.8550 | -21.6 | -11.5 | -44.7 | -21 | -8.8 | -21.1 | -20.3 | -33.6 |  | |
| family | Eubacteriaceae | | 0.5031 | 0.3372 | 0.3166 | 0.4279 | -36.3 | -20.7 | -66.4 | -40 | -15.1 | -26.7 | -28.7 | -50.7 |  | |
| genus | *Eubacterium* | | 0.5028 | 0.3366 | 0.3164 | 0.4275 | -36.3 | -20.6 | -66.4 | -40 | -15.1 | -26.9 | -28.7 | -50.7 |  | |
| **phylum** | **TM7** | | 0.0103 | 0.0285 | 0.3795 | 0.3140 | -10.8 | -30.4 | 33.2 | -59.1 | 25.4 | 53.4 | 24 | 81 |  | |
| **phylum** | **Spirochaetes** | | 0.6829 | 0.7083 | 0.7529 | 0.6971 | 32.2 | 6.7 | 36.6 | -2.9 | 13.2 | 29 | 1.5 | 33.4 |  | |
| class | Spirochaetes | | 0.6829 | 0.7083 | 0.7529 | 0.6971 | 32.2 | 6.7 | 36.6 | -2.9 | 13.2 | 29 | 1.5 | 33.4 |  | |
| order | Spirochaetales | | 0.6829 | 0.7083 | 0.7529 | 0.6971 | 32.2 | 6.7 | 36.6 | -2.9 | 13.2 | 29 | 1.5 | 33.4 |  | |
| family | Spirochaetaceae | | 0.5487 | 0.5597 | 0.7491 | 0.6929 | 32.7 | 4.3 | 38 | -3.1 | 13.3 | 29.1 | 1.7 | 33.7 |  | |
| genus | *Treponema* | | 0.5279 | 0.5378 | 0.7411 | 0.6871 | 33.1 | 4.3 | 38.1 | -3.1 | 14 | 28.8 | 1.9 | 33.6 |  | |
| **phylum** | **Bacteroidetes** | | 16.3960 | 17.2892 | 17.1427 | 15.8709 | 42.2 | 36.2 | 53.7 | 48.5 | 36.5 | 38.5 | 49.4 | 46 |  | |
| class | Bacteroidia | | 16.0344 | 16.7826 | 16.3232 | 14.7560 | 42.3 | 36 | 53.7 | 48.4 | 36.1 | 39.6 | 48.8 | 46.4 |  | |
| order | Bacteroidales | | 16.0344 | 16.7826 | 16.3232 | 14.7560 | 42.3 | 36 | 53.7 | 48.4 | 36.1 | 39.6 | 48.8 | 46.4 |  | |
| family | Prevotellaceae | | 13.8823 | 14.8497 | 15.5044 | 13.6949 | 44.2 | 35.2 | 55.5 | 46.5 | 36.2 | 40.5 | 48.4 | 46.7 |  | |
| genus | *Prevotella* | | 13.4964 | 14.2251 | 15.0931 | 13.2472 | 44.6 | 35.1 | 56 | 46.7 | 36.3 | 40.7 | 48.6 | 47.1 |  | |
| genus | *Hallella* | | 0.0157 | 0.0272 | 0.0440 | 0.0287 | 32.5 | 36.9 | 52.6 | 42.2 | 102.6 | 172 | 207.5 | 248.6 |  | |
| family | Porphyromonadaceae | | 0.9728 | 0.5441 | 0.2855 | 0.4959 | 49.8 | 85.2 | 74.4 | 152 | 39.6 | 25.6 | 72.4 | 38.1 |  | |
| genus | *Tannerella* | | 0.0020 | 0.0008 | 0.0316 | 0.0345 | -5.9 | 49.6 | 8.3 | 21.6 | 20.4 | 23.5 | -26.1 | -10.5 |  | |
| genus | *Porphyromonas* | | 0.8656 | 0.4580 | 0.2098 | 0.4034 | 53.4 | 96.4 | 82.4 | 174.6 | 49.1 | 27.8 | 101.8 | 44.8 |  | |
| class | Flavobacteria | | 0.0704 | 0.0396 | 0.0634 | 0.1027 | 12.6 | 59.8 | 1.6 | 67.8 | 142.4 | 80.2 | 211.6 | 139.1 |  | |
| order | Flavobacteriales | | 0.0704 | 0.0396 | 0.0634 | 0.1027 | 12.6 | 59.8 | 1.6 | 67.8 | 142.4 | 80.2 | 211.6 | 139.1 |  | |
| family | Flavobacteriaceae | | 0.0704 | 0.0396 | 0.0611 | 0.0993 | 11.6 | 58.4 | 0.8 | 66.9 | 141.7 | 80.7 | 214.1 | 139.5 |  | |
| **phylum** | **Proteobacteria** | | 6.1708 | 6.4873 | 6.7424 | 6.5114 | 7.5 | 14.4 | 2.6 | 3.1 | 20.6 | 19.1 | 10.5 | 20.1 |  | |
| class | Epsilonproteobacteria | | 4.0890 | 3.6428 | 3.9992 | 4.3657 | 5 | 16.8 | -2 | -1.3 | 21 | 6.3 | 4.4 | 1.4 |  | |
| order | Campylobacterales | | 4.0676 | 3.6114 | 3.9856 | 4.3487 | 5 | 16.8 | -2.1 | -1.3 | 21 | 6.3 | 4.5 | 1.4 |  | |
| family | Campylobacteraceae | | 3.9879 | 3.5293 | 3.9666 | 4.3166 | 5.1 | 16.5 | -2.3 | -1.3 | 21 | 6.4 | 4.5 | 1.4 |  | |
| genus | *Campylobacter* | | 3.9830 | 3.5195 | 3.9575 | 4.3063 | 5.1 | 16.5 | -2.3 | -1.3 | 21.1 | 6.4 | 4.7 | 1.4 |  | |
| class | Deltaproteobacteria | | 0.0091 | 0.0086 | 0.0090 | 0.0100 | -33.4 | -30.6 | -23.8 | -42.9 | 2.2 | -33.1 | -0.6 | -21.2 |  | |
| class | Betaproteobacteria | | 1.1733 | 1.0125 | 0.9862 | 1.1632 | 13.7 | 15 | 11 | 18.9 | 9.2 | 18.7 | 4.4 | 22.7 |  | |
| order | Burkholderiales | | 0.0014 | 0.0015 | 0.0111 | 0.0168 | 54.1 | 74.6 | 76.1 | 45.9 | 28.9 | 21.8 | 65.8 | 48 |  | |
| order | Neisseriales | | 1.1242 | 0.9577 | 0.9032 | 1.0443 | 13.7 | 14.2 | 10.6 | 17.5 | 9.8 | 18.7 | 4.8 | 22.9 |  | |
| family | Neisseriaceae | | 1.1242 | 0.9577 | 0.9032 | 1.0443 | 13.7 | 14.2 | 10.6 | 17.5 | 9.8 | 18.7 | 4.8 | 22.9 |  | |
| genus | *Neisseria* | | 0.9253 | 0.7584 | 0.8070 | 0.9427 | 14.1 | 14.5 | 9.8 | 15.2 | 12.2 | 20.3 | 7.7 | 24.6 |  | |
| genus | *Kingella* | | 0.0255 | 0.0238 | 0.0625 | 0.0532 | -23.8 | -14.4 | -1 | -21.9 | -23.2 | -1.6 | -29.8 | 9 |  | |
| class | Gammaproteobacteria | | 0.3618 | 0.9654 | 1.5587 | 0.7574 | 19.9 | 3.8 | 28.3 | 0.5 | 29.1 | 91.8 | 32.2 | 123.6 |  | |
| order | Pasteurellales | | 0.2134 | 0.4817 | 1.5054 | 0.7105 | 25.5 | 2.8 | 35.9 | 0.9 | 29 | 91.5 | 32.9 | 124.8 |  | |
| family | Pasteurellaceae | | 0.2134 | 0.4817 | 1.5054 | 0.7105 | 25.5 | 2.8 | 35.9 | 0.9 | 29 | 91.5 | 32.9 | 124.8 |  | |
| order | Cardiobacteriales | | 0.0066 | 0.0028 | 0.0136 | 0.0147 | 18.5 | 79.6 | 8.3 | 107.5 | 2.9 | 27.3 | -5.7 | 5.7 |  | |
| family | Cardiobacteriaceae | | 0.0066 | 0.0028 | 0.0136 | 0.0147 | 18.5 | 79.6 | 8.3 | 107.5 | 2.9 | 27.3 | -5.7 | 5.7 |  | |
| genus | *Cardiobacterium* | | 0.0066 | 0.0028 | 0.0136 | 0.0147 | 18.5 | 79.6 | 8.3 | 107.5 | 2.9 | 27.3 | -5.7 | 5.7 |  | |
| **phylum** | **Cyanobacteria** | | 0.0113 | 0.0081 | 0.0113 | 0.0158 | 47.2 | 32.2 | 31 | 47 | 39.4 | 30.3 | 19.7 | 23.6 |  | |
| class | Cyanobacteria | | 0.0113 | 0.0081 | 0.0113 | 0.0158 | 47.2 | 32.2 | 31 | 47 | 39.4 | 30.3 | 19.7 | 23.6 |  | |
| family | Chloroplast | | 0.0106 | 0.0069 | 0.0113 | 0.0158 | 35.3 | 37.5 | 25.5 | 57.7 | 39.4 | 29.1 | 19.7 | 23.6 |  | |
| genus | *Streptophyta* | | 0.0103 | 0.0066 | 0.0109 | 0.0153 | 38.1 | 39.7 | 22.6 | 61.2 | 42.3 | 28.9 | 20.3 | 21.6 |  | |
| **phylum** | **Tenericutes** | | 0.1362 | 0.0816 | 0.0759 | 0.1336 | 32.7 | 36.9 | 85.7 | 139 | 22.7 | 24.3 | 125.5 | 82.6 |  | |
| class | Mollicutes | | 0.1362 | 0.0816 | 0.0759 | 0.1336 | 32.7 | 36.9 | 85.7 | 139 | 22.7 | 24.3 | 125.5 | 82.6 |  | |
| order | Mycoplasmatales | | 0.1361 | 0.0816 | 0.0759 | 0.1323 | 32.8 | 36.9 | 85.9 | 139 | 22.2 | 24.3 | 124.6 | 83.3 |  | |
| family | Mycoplasmataceae | | 0.1361 | 0.0816 | 0.0759 | 0.1323 | 32.8 | 36.9 | 85.9 | 139 | 22.2 | 24.3 | 124.6 | 83.3 |  | |
| genus | *Mycoplasma* | | 0.1361 | 0.0816 | 0.0759 | 0.1323 | 32.8 | 36.9 | 85.9 | 139 | 22.2 | 24.3 | 124.6 | 83.3 |  | |
| **phylum** | **SR1** | | 0.0120 | 0.0220 | 0.0793 | 0.0671 | 30.2 | 0.6 | 104.5 | -7.7 | 33.8 | 23.3 | 11.9 | 53.1 |  | |
| **phylum** | **Fusobacteria** | | 2.8905 | 2.7217 | 2.8748 | 3.0147 | 22.6 | 15.5 | 10.6 | 4.6 | 14.5 | 33.6 | -4.1 | 60.9 |  | |
| class | Fusobacteria | | 2.8905 | 2.7217 | 2.8748 | 3.0147 | 22.6 | 15.5 | 10.6 | 4.6 | 14.5 | 33.6 | -4.1 | 60.9 |  | |
| order | Fusobacteriales | | 2.8905 | 2.7217 | 2.8748 | 3.0147 | 22.6 | 15.5 | 10.6 | 4.6 | 14.5 | 33.6 | -4.1 | 60.9 |  | |
| family | Leptotrichiaceae | | 0.3527 | 0.2702 | 0.4100 | 0.5135 | 26.4 | 17.2 | 41.4 | 34.2 | 10.9 | 23.6 | 6.9 | 33.9 |  | |
| genus | *Leptotrichia* | | 0.3155 | 0.2383 | 0.4031 | 0.5014 | 25.6 | 20.7 | 40.3 | 36.3 | 10.6 | 24 | 6.7 | 33.7 |  | |
| family | Fusobacteriaceae | | 2.4475 | 2.3807 | 2.4289 | 2.4416 | 21.5 | 15.1 | 5.3 | 0.3 | 14.7 | 35.4 | -6.3 | 67.3 |  | |
| genus | *Fusobacterium* | | 2.4220 | 2.3318 | 2.3994 | 2.4060 | 21.1 | 14.6 | 4.7 | -0.3 | 14.7 | 35.3 | -6.2 | 67.6 |  | |

^a^ ((Proportion in 25- or 30-cycle subset) - (proportion in 20 cycle-subset))/(proportion in 20-cycle subset) x 100.

B

|  |  |  | **Frequency (%)** | | **Change in OTU frequency** | | | |  |
| --- | --- | --- | --- | --- | --- | --- | --- | --- | --- |
|  |  |  | **F** | **R** | **F** | **R** | **F** | **R** | **Run** |
|  |  |  | **20** | **20** | **25** | **25** | **30** | **30** | **Cycles** |
| **Phylum** | **OTU #** |  | **V1 region** | | | | | |  |
| Actinobacteria | 1 | | 1.8946 | 2.0569 | -7.4 | -10.9 | -28.7 | -26.1 |  |
|  | 2 | | 1.8237 | 0.7188 | -7.6 | -10.6 | -27.9 | -24.6 |  |
|  | 3 | | 0.9250 | 0.4319 | -15.5 | -1.9 | -28.7 | -5.8 |  |
|  | 4 | | 0.7759 | 0.7731 | -12.1 | -15.7 | -31.8 | -30.8 |  |
|  | 5 | | 0.3280 | 0.0796 | -8.6 | 18.8 | -28.5 | 8.0 |  |
|  | 6 | | 0.1923 | 0.0719 | -23.7 | -14.3 | -30.6 | -28.6 |  |
|  | 7 | | 0.1889 | 0.0599 | -9.6 | -3.1 | -18.4 | -10.4 |  |
|  | 8 | | 0.1803 | 0.0814 | -0.1 | 5.0 | -28.8 | 8.5 |  |
|  | 9 | | 0.1602 | 0.0895 | -4.8 | 1.9 | -27.3 | -8.0 |  |
|  | 10 | | 0.1486 | 0.0681 | -7.9 | 0.6 | -22.1 | 7.7 |  |
|  | 11 | | 0.1283 | 0.0313 | 0.1 | -15.2 | -21.6 | 1.9 |  |
|  | 12 | | 0.0969 | 0.1319 | -7.8 | -21.8 | -33.4 | -32.6 |  |
|  | 13 | | 0.0439 | 0.1512 | -16.6 | -13.4 | -43.3 | -24.2 |  |
|  | 14 | | 0.0311 | 0.1175 | -12.9 | -15.4 | -31.1 | -27.6 |  |
| Firmicutes | 15 | | 9.3404 | 7.0382 | -27.6 | -19.6 | -37.7 | -31.0 |  |
|  | 16 | | 8.7145 | 8.9814 | -16.8 | -19.3 | -12.2 | -22.6 |  |
|  | 17 | | 5.9782 | 5.7623 | -15.3 | -20.1 | -19.0 | -23.4 |  |
|  | 18 | | 5.2846 | 6.0438 | -8.3 | -16.4 | -3.1 | -15.2 |  |
|  | 19 | | 5.2536 | 5.3525 | -4.6 | -17.1 | 0.6 | -14.0 |  |
|  | 20 | | 3.0651 | 2.0815 | -10.3 | 1.2 | -11.0 | 6.5 |  |
|  | 21 | | 2.5390 | 1.2838 | -14.8 | 5.2 | -26.0 | 1.5 |  |
|  | 22 | | 1.2795 | 1.0219 | -10.6 | -14.9 | -10.5 | -22.7 |  |
|  | 23 | | 0.9427 | 0.3702 | -17.2 | 1.5 | -25.1 | 1.6 |  |
|  | 24 | | 0.9319 | 1.2422 | -19.5 | -16.7 | -16.7 | -26.7 |  |
|  | 25 | | 0.8647 | 0.5747 | -21.5 | -11.4 | -44.6 | -19.2 |  |
|  | 26 | | 0.7869 | 0.8053 | -7.6 | -18.2 | -0.6 | -12.8 |  |
|  | 27 | | 0.6859 | 0.7723 | -19.0 | -16.2 | -14.8 | -20.8 |  |
|  | 28 | | 0.6512 | 0.7158 | -7.1 | -9.1 | 6.7 | -0.4 |  |
|  | 29 | | 0.6058 | 0.2696 | -27.9 | -12.5 | -61.1 | -20.9 |  |
|  | 30 | | 0.5229 | 0.6213 | -15.9 | -14.0 | -24.8 | -19.5 |  |
|  | 31 | | 0.5046 | 0.6616 | -14.6 | -25.1 | -20.4 | -24.2 |  |
|  | 32 | | 0.4572 | 0.6222 | -34.2 | -23.6 | -40.4 | -33.0 |  |
|  | 33 | | 0.4185 | 0.6034 | -11.2 | -13.2 | -1.3 | -13.1 |  |
|  | 34 | | 0.3873 | 0.5241 | -9.6 | -17.2 | -4.0 | -19.0 |  |
|  | 35 | | 0.3660 | 0.5017 | -14.8 | -20.9 | -13.2 | -19.9 |  |
|  | 36 | | 0.3245 | 0.4462 | -17.5 | -18.2 | -19.8 | -22.9 |  |
|  | 37 | | 0.2803 | 0.2603 | -19.3 | -16.9 | -20.1 | -22.0 |  |
|  | 38 | | 0.2586 | 0.1728 | -40.6 | -20.1 | -69.4 | -36.4 |  |
|  | 39 | | 0.2578 | 0.1197 | -20.5 | -26.7 | -43.7 | -43.9 |  |
|  | 40 | | 0.2554 | 0.2295 | -22.4 | -20.3 | -25.5 | -29.1 |  |
|  | 41 | | 0.2401 | 0.3312 | -21.2 | -22.0 | -18.9 | -25.8 |  |
|  | 42 | | 0.2355 | 0.1302 | -16.1 | 7.2 | -33.3 | -4.6 |  |
|  | 43 | | 0.2250 | 0.2516 | 58.0 | 41.9 | 142.5 | 88.5 |  |
|  | 44 | | 0.2114 | 0.3479 | -12.0 | -16.4 | -11.4 | -22.0 |  |
|  | 45 | | 0.2095 | 0.1931 | -5.0 | 11.0 | -12.8 | 16.2 |  |
|  | 46 | | 0.2063 | 0.1079 | -22.4 | -12.2 | -32.8 | -24.7 |  |
|  | 47 | | 0.1935 | 0.3143 | -8.0 | -19.1 | -12.7 | -14.8 |  |
|  | 48 | | 0.1909 | 0.3430 | -2.5 | -18.8 | 5.2 | -17.4 |  |
|  | 49 | | 0.1886 | 0.1447 | -32.5 | -27.2 | -49.1 | -43.7 |  |
|  | 50 | | 0.1695 | 0.3130 | -6.6 | -12.5 | 1.0 | -14.2 |  |
|  | 51 | | 0.1693 | 0.1893 | 2.5 | 17.7 | -0.2 | 17.7 |  |
|  | 52 | | 0.1442 | 0.1000 | -9.3 | 10.4 | -20.2 | 25.5 |  |
|  | 53 | | 0.1410 | 0.2888 | -33.2 | -18.6 | -43.0 | -28.3 |  |
|  | 54 | | 0.1349 | 0.1479 | -29.6 | -24.2 | -40.1 | -22.7 |  |
|  | 55 | | 0.1332 | 0.1049 | -17.7 | 1.7 | -26.1 | 7.9 |  |
|  | 56 | | 0.1248 | 0.0576 | -23.1 | 14.1 | -39.0 | 9.6 |  |
|  | 57 | | 0.1238 | 0.1053 | -14.1 | -26.4 | -29.6 | -38.1 |  |
|  | 58 | | 0.1087 | 0.1906 | -14.0 | -23.4 | -7.5 | -27.2 |  |
|  | 59 | | 0.1047 | 0.0674 | -15.7 | 22.8 | -20.5 | 22.7 |  |
|  | 60 | | 0.0844 | 0.1441 | -27.3 | -18.0 | -39.5 | -28.5 |  |
|  | 61 | | 0.0770 | 0.1009 | 6.8 | 8.7 | 5.7 | 17.0 |  |
|  | 62 | | 0.0756 | 0.1216 | -27.6 | -18.7 | -34.9 | -33.8 |  |
|  | 63 | | 0.0628 | 0.1021 | -31.3 | -23.7 | -43.2 | -23.8 |  |
|  | 64 | | 0.0517 | 0.1141 | -7.7 | 2.5 | -10.4 | 1.9 |  |
|  | 65 | | 0.0420 | 0.1488 | -30.7 | -17.2 | -26.5 | -21.6 |  |
|  | 66 | | 0.0397 | 0.1567 | -20.9 | -28.4 | -28.1 | -22.1 |  |
| Spirochaetes | 67 | | 0.1241 | 0.1507 | 21.0 | 4.4 | 19.7 | -10.1 |  |
| Bacteroidetes | 68 | | 5.7264 | 5.6571 | 51.5 | 35.2 | 65.8 | 45.9 |  |
|  | 69 | | 2.3275 | 2.1034 | 46.3 | 34.7 | 57.8 | 49.7 |  |
|  | 70 | | 1.9363 | 1.7570 | 40.5 | 36.3 | 49.9 | 38.2 |  |
|  | 71 | | 0.6100 | 0.5228 | 23.4 | 19.0 | 26.6 | 21.6 |  |
|  | 72 | | 0.5354 | 0.3400 | 17.5 | 40.9 | 14.8 | 60.0 |  |
|  | 73 | | 0.3942 | 0.1843 | 59.9 | 104.7 | 80.8 | 188.2 |  |
|  | 74 | | 0.2512 | 0.2342 | 9.8 | 1.0 | -12.0 | -26.4 |  |
|  | 75 | | 0.2281 | 0.0854 | 37.2 | 73.0 | 65.6 | 124.6 |  |
|  | 76 | | 0.2252 | 0.1724 | 15.6 | 73.0 | 19.6 | 63.4 |  |
|  | 77 | | 0.2056 | 0.1554 | -3.7 | 7.1 | -7.8 | -0.6 |  |
|  | 78 | | 0.1965 | 0.3865 | 52.1 | 35.3 | 52.8 | 50.3 |  |
|  | 79 | | 0.1504 | 0.1041 | 11.9 | 3.5 | -8.3 | -2.9 |  |
|  | 80 | | 0.1391 | 0.1599 | 30.6 | 23.4 | 37.9 | 32.1 |  |
|  | 81 | | 0.1386 | 0.1786 | 41.7 | 35.9 | 61.7 | 52.3 |  |
|  | 82 | | 0.1308 | 0.2520 | 40.7 | 41.3 | 49.9 | 58.7 |  |
|  | 83 | | 0.1177 | 0.0822 | 20.4 | 15.5 | 12.5 | 24.6 |  |
|  | 84 | | 0.1055 | 0.1156 | 48.2 | 29.4 | 58.1 | 43.4 |  |
|  | 85 | | 0.1001 | 0.0679 | 56.7 | 70.0 | 86.5 | 140.4 |  |
|  | 86 | | 0.0780 | 0.2126 | 57.6 | 24.9 | 64.6 | 54.3 |  |
|  | 87 | | 0.0454 | 0.1163 | 68.4 | 43.2 | 81.6 | 61.4 |  |
|  | 88 | | 0.0452 | 0.1120 | 42.2 | 45.5 | 61.3 | 54.6 |  |
|  | 89 | | 0.0420 | 0.1131 | 56.3 | 32.4 | 59.3 | 54.0 |  |
|  | 90 | | 0.0351 | 0.1019 | 39.2 | 17.8 | 43.2 | 32.2 |  |
| Proteobacteria | 91 | | 3.6671 | 3.2985 | 3.6 | 13.8 | -4.0 | -5.0 |  |
|  | 92 | | 0.4563 | 0.3229 | -35.8 | -28.2 | -75.5 | -58.4 |  |
|  | 93 | | 0.3927 | 0.2914 | -5.4 | 7.9 | -27.6 | -13.4 |  |
|  | 94 | | 0.3837 | 0.3034 | 6.5 | 13.3 | 0.5 | 7.0 |  |
|  | 95 | | 0.3436 | 0.2203 | 9.8 | 10.1 | 4.7 | 10.9 |  |
|  | 96 | | 0.1210 | 0.1062 | -32.7 | -24.2 | -74.3 | -60.7 |  |
|  | 97 | | 0.1072 | 0.2375 | 3.5 | -9.8 | 6.8 | -11.1 |  |
|  | 98 | | 0.1040 | 0.0737 | -3.8 | 8.9 | -5.4 | -21.1 |  |
|  | 99 | | 0.0611 | 0.1103 | 0.4 | 26.6 | -0.3 | 0.2 |  |
|  | 100 | | 0.0542 | 0.1578 | 13.3 | -3.0 | 15.1 | -12.8 |  |
|  | 101 | | 0.0410 | 0.1105 | -4.9 | -4.8 | 4.3 | -8.4 |  |
| Cyanobacteria | 102 | | 0.1200 | 0.0561 | -23.6 | 9.4 | -61.6 | -31.4 |  |
| Fusobacteria | 103 | | 1.4631 | 1.2037 | 14.4 | 7.8 | -11.6 | -12.5 |  |
|  | 104 | | 0.4185 | 0.3150 | 30.1 | 18.7 | 22.2 | 11.3 |  |
|  | 105 | | 0.1952 | 0.1524 | 25.5 | 4.6 | 14.4 | -2.7 |  |
|  | 106 | | 0.1254 | 0.0702 | 26.2 | 8.0 | 38.4 | 27.4 |  |
|  |  | | **V3 region** | | | | | |  |
| Actinobacteria | 1 | | 3.7305 | 3.6188 | -8.6 | -5.8 | -16.1 | -30.4 |  |
|  | 2 | | 0.7849 | 0.9989 | 0.0 | -1.4 | 8.8 | -15.6 |  |
|  | 3 | | 0.4690 | 0.4667 | 3.0 | -4.1 | -17.6 | -32.1 |  |
|  | 4 | | 0.1868 | 0.1930 | -8.9 | -12.7 | -7.3 | -17.0 |  |
|  | 5 | | 0.1257 | 0.1975 | 8.9 | -7.5 | 5.9 | -28.2 |  |
| Firmicutes | 6 | | 34.9812 | 28.0275 | -15.1 | -13.3 | -13.2 | -10.9 |  |
|  | 7 | | 6.4961 | 6.5394 | -12.8 | -24.8 | -21.2 | -35.4 |  |
|  | 8 | | 3.0097 | 4.5885 | -1.1 | -18.6 | -5.3 | -28.1 |  |
|  | 9 | | 2.0358 | 2.1711 | -7.5 | -12.5 | -3.4 | -15.2 |  |
|  | 10 | | 1.8063 | 1.8364 | -15.8 | -22.6 | -22.2 | -37.5 |  |
|  | 11 | | 0.9112 | 0.9947 | -33.4 | -17.0 | -39.3 | -10.6 |  |
|  | 12 | | 0.8548 | 1.0139 | -20.5 | -18.8 | -32.6 | -28.2 |  |
|  | 13 | | 0.8003 | 0.5802 | -25.3 | -17.2 | -24.6 | -23.2 |  |
|  | 14 | | 0.4683 | 0.5178 | -16.5 | -27.4 | -46.0 | -58.3 |  |
|  | 15 | | 0.4257 | 0.4454 | -8.4 | -19.2 | -19.6 | -32.0 |  |
|  | 16 | | 0.3210 | 0.3238 | -23.3 | -24.1 | -42.2 | -43.7 |  |
|  | 17 | | 0.3184 | 0.5546 | -11.0 | -19.1 | -6.1 | -40.8 |  |
|  | 18 | | 0.3020 | 0.3240 | -18.0 | -23.3 | -32.5 | -39.5 |  |
|  | 19 | | 0.2854 | 0.3274 | -30.9 | -14.6 | -41.0 | -15.1 |  |
|  | 20 | | 0.2811 | 0.3252 | -20.5 | -19.2 | -29.9 | -33.8 |  |
|  | 21 | | 0.2801 | 0.5538 | -17.9 | -21.3 | -35.0 | -30.5 |  |
|  | 22 | | 0.2629 | 0.2428 | -14.5 | -21.6 | -12.4 | -23.6 |  |
|  | 23 | | 0.2423 | 0.2162 | -9.4 | -14.1 | -13.2 | -11.7 |  |
|  | 24 | | 0.2188 | 0.2075 | 10.2 | -15.1 | -10.7 | -11.1 |  |
|  | 25 | | 0.2055 | 0.1642 | 12.6 | 17.0 | 36.6 | 26.4 |  |
|  | 26 | | 0.2047 | 0.2907 | -13.6 | -30.8 | -24.1 | -36.1 |  |
|  | 27 | | 0.1799 | 0.1972 | -10.8 | -8.9 | -10.4 | -23.2 |  |
|  | 28 | | 0.1651 | 0.1521 | -0.3 | -2.7 | -24.4 | -20.1 |  |
|  | 29 | | 0.1448 | 0.1301 | -29.1 | -7.0 | -19.5 | -18.7 |  |
|  | 30 | | 0.1326 | 0.1749 | -19.1 | -27.1 | -27.2 | -52.8 |  |
|  | 31 | | 0.1275 | 0.1255 | -35.0 | -7.5 | -49.9 | -10.5 |  |
|  | 32 | | 0.1206 | 0.1557 | -21.6 | -28.2 | -32.4 | -35.7 |  |
|  | 33 | | 0.1174 | 0.1188 | -26.9 | -10.9 | -26.6 | -10.6 |  |
|  | 34 | | 0.1128 | 0.1172 | -20.0 | -1.9 | 1.0 | -2.6 |  |
|  | 35 | | 0.1100 | 0.1393 | -27.1 | -21.2 | -41.4 | -13.2 |  |
|  | 36 | | 0.0932 | 0.1936 | -17.6 | -17.6 | -26.7 | -24.2 |  |
|  | 37 | | 0.0906 | 0.1099 | -20.4 | -24.2 | -35.6 | -46.7 |  |
|  | 38 | | 0.0899 | 0.1122 | -9.4 | -9.4 | -23.2 | -32.3 |  |
|  | 39 | | 0.0404 | 0.1463 | -4.1 | -26.2 | -6.6 | -36.7 |  |
|  | 40 | | 0.0471 | 0.1321 | -14.4 | -14.6 | -26.6 | -37.7 |  |
|  | 41 | | 0.0348 | 0.1065 | -2.8 | -26.1 | -19.7 | -41.4 |  |
|  | 42 | | 0.0489 | 0.1020 | 30.3 | 2.1 | 52.6 | 2.9 |  |
| TM7 | 43 | | 0.1948 | 0.2217 | -0.6 | -3.9 | -16.7 | -1.9 |  |
| Spirochaetes | 44 | | 0.1557 | 0.1408 | 10.2 | 31.8 | 5.5 | 34.4 |  |
|  | 45 | | 0.1312 | 0.1184 | 12.3 | 26.5 | -1.4 | 15.6 |  |
|  | 46 | | 0.1204 | 0.1095 | 19.5 | 23.8 | -5.8 | 22.9 |  |
|  | 47 | | 0.1146 | 0.1003 | 4.6 | 16.2 | -8.8 | 30.6 |  |
|  | 48 | | 0.1012 | 0.0939 | 19.2 | 30.2 | 2.4 | 31.3 |  |
| Bacteroidetes | 49 | | 5.9256 | 4.9095 | 36.6 | 44.7 | 53.4 | 50.2 |  |
|  | 50 | | 3.0953 | 2.5726 | 31.5 | 43.9 | 44.1 | 50.1 |  |
|  | 51 | | 1.2544 | 1.1500 | 34.3 | 32.0 | 26.4 | 40.7 |  |
|  | 52 | | 0.6306 | 0.4434 | 42.3 | 49.5 | 71.4 | 74.1 |  |
|  | 53 | | 0.5933 | 0.6041 | 23.2 | 23.2 | 34.8 | 22.2 |  |
|  | 54 | | 0.5832 | 0.5700 | 27.4 | 30.0 | 49.4 | 36.2 |  |
|  | 55 | | 0.3961 | 0.3819 | 21.8 | -2.1 | 0.9 | 11.2 |  |
|  | 56 | | 0.3694 | 0.3261 | -2.8 | 27.9 | 7.9 | 23.6 |  |
|  | 57 | | 0.3502 | 0.3365 | 15.1 | 17.5 | -14.9 | 8.3 |  |
|  | 58 | | 0.2942 | 0.4147 | 28.4 | 8.1 | 41.2 | 10.9 |  |
|  | 59 | | 0.2892 | 0.2835 | 87.0 | 38.0 | 123.9 | 55.4 |  |
|  | 60 | | 0.2108 | 0.1717 | 68.9 | 42.1 | 65.2 | 48.1 |  |
|  | 61 | | 0.1697 | 0.1297 | 30.5 | 48.3 | 50.3 | 62.0 |  |
|  | 62 | | 0.1679 | 0.1864 | 11.8 | 7.1 | 0.7 | 0.7 |  |
|  | 63 | | 0.1570 | 0.2760 | 28.5 | 18.2 | 83.3 | 39.2 |  |
|  | 64 | | 0.1527 | 0.1274 | 45.8 | 11.8 | 62.0 | 77.4 |  |
|  | 65 | | 0.1275 | 0.1029 | 41.5 | 41.0 | 28.1 | 23.9 |  |
|  | 66 | | 0.1070 | 0.1171 | 36.8 | 31.4 | 62.5 | 22.4 |  |
|  | 67 | | 0.0945 | 0.1619 | 58.9 | 27.6 | 110.4 | 41.8 |  |
| Fusobacteria | 68 | | 2.1970 | 2.0727 | 13.9 | 35.2 | -6.8 | 73.1 |  |
|  | 69 | | 0.1817 | 0.2060 | 5.9 | 15.0 | 3.0 | 22.9 |  |
|  | 70 | | 0.1164 | 0.1314 | 11.7 | 33.0 | -2.7 | 50.4 |  |
| Proteobacteria | 71 | | 3.5451 | 3.7025 | 19.6 | 5.3 | 3.7 | 1.0 |  |
|  | 72 | | 1.1893 | 0.5363 | 24.3 | 84.6 | 31.7 | 115.5 |  |
|  | 73 | | 0.6103 | 0.6330 | 11.8 | 21.6 | 10.1 | 26.4 |  |
|  | 74 | | 0.1944 | 0.0818 | 29.1 | 98.5 | 17.6 | 142.2 |  |
|  | 75 | | 0.1072 | 0.0826 | -6.0 | -0.5 | -21.0 | 11.5 |  |
|  | 76 | | 0.0904 | 0.1154 | 11.6 | 6.1 | -10.5 | -16.6 |  |
| Tenericutes | 77 | | 0.0666 | 0.1139 | 21.3 | 17.4 | 129.5 | 82.2 |  |
